# Obstetrician–Gynecologists in General Practice in New Mexico: A Comparison Between Rural and Metropolitan Counties

**DOI:** 10.1089/whr.2020.0070

**Published:** 2020-10-14

**Authors:** William F. Rayburn, Trevor E. Quiner, Jacquelyn A. Blackstone

**Affiliations:** Department of Obstetrics and Gynecology, University of New Mexico School of Medicine, Albuquerque, New Mexico, USA.

**Keywords:** gynecology, obstetrics, physicians', practice patterns, rural health, surveys

## Abstract

***Objective:*** About one-half of all U.S. counties lack obstetrician–gynecologist (ob-gyns) physicians especially in rural areas. The objective of this study was to use experience in our state to identify demographic and practice characteristics distinguishing ob-gyns in general practice (general ob-gyns) in rural and metropolitan settings.

***Materials and Methods:*** This retrospective observational study used self-reported responses by physicians to a mandated survey about demographics and practice patterns at the time of New Mexico medical relicensing. Included in the study were all general ob-gyns in 2016 and 2017. Information about subspecialist ob-gyns and residents who graduated that year was obtained from the American College of Obstetricians and Gynecologists and the Accreditation Council for Graduate Medical Education from 2016 to 2019.

***Results:*** Nearly 1 in 3 (84 of 273, 30.8%) general ob-gyns practiced in a rural county. Those in rural settings tended to be older (*p* = 0.02) and male (*p* = 0.04). Most had practices in both obstetrics and gynecology. Compared with those in metropolitan counties, general ob-gyns in rural counties practiced in smaller groups (*p* = 0.0003) and worked 40 hours or more weekly (*p* = 0.0003). All subspecialists practiced in the most populous metropolitan county. No recent residency graduate practiced rurally in New Mexico.

***Conclusions:*** General ob-gyns in New Mexico's rural counties practiced in smaller groups and for longer work hours. Rural ob-gyns tended to be older and male.

## Introduction

The United States is predicted to face a shortage of 46,900 to 121,900 physicians by 2032.^1^ This problem is evident not only for primary care physicians but also for medical specialists and surgeons. By 2020, it is estimated that the United States will have a shortage of 6,000–8,800 obstetricians–gynecologists (ob-gyns) with a reported increase in that shortage to 22,000 by 2050.^2^ Furthermore, a geographic maldistribution of ob-gyns has received consideration, particularly as fewer family physicians provide maternity care and rural hospitals threaten to discontinue obstetric services due to cost reduction.^3–5^

General ob-gyns are essential as frontline caregivers of adult women, who constitute about two-fifths of the total U.S. population.^6^ The greatest need for women's health care will be in states where population growth is highest, the number of Hispanic women is increasing, the supply of ob-gyns is already suboptimal, and driving times to subspecialists and tertiary care centers are longer.^6^ New Mexico meets three of these benchmarks. Despite a statewide increase in ob-gyns between 1990 and 2014, the median number of ob-gyns per 10,000 women in 2018 lagged behind in rural counties than in the state's most populous county (5.6 vs. 9.7).^7^ Bernallilo County is home to one-third of New Mexico's adult females, the University of New Mexico (UNM) School of Medicine, and where many ob-gyns, like other medical specialists, frequently chose to practice.^8^

A key to understanding this statewide maldistribution is by evaluating differences in ob-gyn practices in rural and metropolitan settings. The objective of this study was to examine the demographics and practice patterns of New Mexico nonsubspecialist (general) ob-gyns to better understand similarities and differences between those in rural and metropolitan settings. These details may assist policy makers and health system administrators in finding solutions to community and women's health care needs in under-resourced rural areas.^9^

## Materials and Methods

To facilitate evidence-based solutions to health care workforce challenges, New Mexico enacted the Health Care Work Force Data Collection, Analysis and Policy Act in 2011.^10^ Under this act, all health care professional licensing boards, including the New Mexico Medical Board, required physicians to complete a demographic and practice survey at license renewal. This retrospective observational study used data from this 70-item survey required for physicians (MD or DO) to take every 3 years for license. Survey items included physicians' self-reported demographics (gender, race, and ethnicity), practice setting (such as outpatient, inpatient, or combined inpatient/outpatient), practice size, hours worked weekly (40 hours or more), and weeks worked annually (40 weeks or more).

Data were obtained from the New Mexico Regulation and Licensing Department (RLD). Licensure rolls were used to identify physicians with a valid license at any point between January 1, 2016, and December 31, 2017. For each active physician, licensure data including date of birth were merged with the individual's current relicensing survey. Ob-gyns were identified by self-reported specialty; age was calculated as of July 1, 2017, using date of birth. The ZIP Code was to be the same as the primary practice site. No practitioner was identified by name, and the study was deemed exempt by our institutional review board, the UNM Human Research Review Committee (registration number: HRRC 13-329).

The physician's scope of practice was self-reported as being either obstetrics or gynecology only (limited) or both (full). Excluded were ob-gyns who were residents in training or subspecialists as listed in New Mexico Section, American College of Obstetricians and Gynecologists membership data from 2016 to 2019. Subspecialists were those completing American Board of Obstetrics and Gynecology-accredited fellowship training for maternal–fetal medicine, gynecologic oncology, female pelvic medicine and reconstructive surgery, or reproductive endocrinology and infertility. The state's single ob-gyn residency program, located at the UNM School of Medicine in Albuquerque, graduated six seniors annually between 2016 and 2019. The relocation of each resident graduating that year was reported by the Accreditation Council for Graduate Medical Education.^11^

Counties were chosen to be the geographic unit of measurement. Demographic and practice data for general ob-gyns practicing in New Mexico during 2016 or 2017 were compared for rural (*n* = 26) or metropolitan (*n* = 7) counties. Rural counties were those identified as nonmetropolitan in the Office of Rural Health Policy list of rural counties, according to the 2010 census for each county.^12^ The Student *t*-test, chi-square test, and Fisher exact test were utilized as appropriate in R version 3.4.2 (The R Foundation, Vienna, Austria).^13^ The significance threshold was set lower (at *p* ≤ 0.005 using the Bonferroni correction for multiple comparisons) to minimize data from incorrectly appearing to be statistically significant.

## Results

**FIG. 1.**
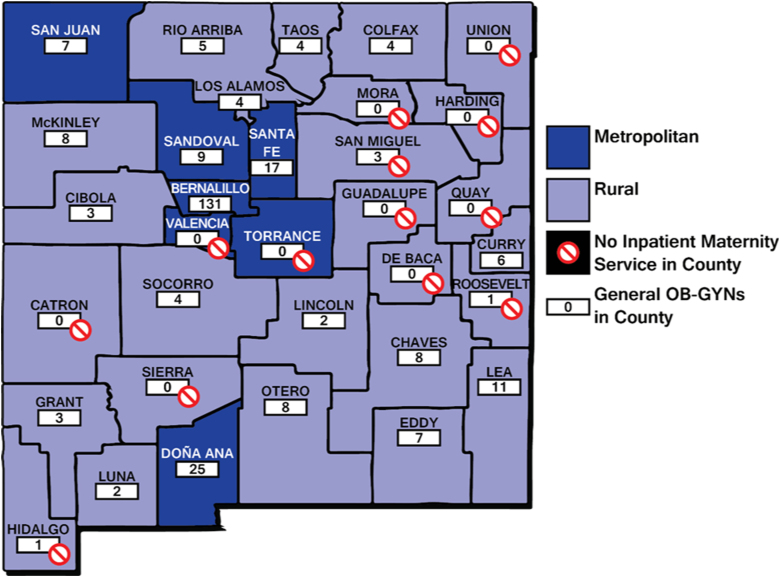
General ob-gyns practicing in rural (light) or metropolitan (dark) counties. “No” symbols indicate counties without inpatient maternity services. ob-gyns, obstetrician–gynecologist.

A total of 273 licensed general ob-gyns were identified in New Mexico between 2016 and 2017. Figure 1 shows their distribution by practice county: nearly 1 in 3 (84, 30.8%) practiced in rural counties. Only 5 (1.9% of total) worked in the 13 counties that had no hospital with a maternity service: 3 in San Miguel county where the maternity service closure was temporary and 2 working on a short-term or part-time basis in either Hidalgo or Roosevelt counties.

Table 1 shows the demographic and practice differences of general ob-gyns, according to their survey responses. As not all questions were answered by surveillants, there are some differences in the totals in each category. General ob-gyns in rural counties tended to be older, with a mean age 3.5 years older than metropolitan practitioners (*p* = 0.02). Rural general ob-gyns tended to be male (54.3% vs. 38.7%; *p* = 0.04). Most general ob-gyns identified themselves as non-Hispanic white, regardless of county. Compared with general ob-gyns in metropolitan counties, those in rural counties had significantly smaller group practices (usually four physicians or fewer; *p* = 0.0003; Fisher's exact test), and all worked 40 hours or more weekly (*p* = 0.002). Regardless of county, nearly all general ob-gyns practiced the full scope of outpatient and inpatient obstetrics and gynecology (95.2% in both metropolitan and rural settings). None practiced solely in a hospital.

**Table 1.** Demographics and Practice Characteristics of Obstetrician–Gynecologist in General Practice in Rural and Metropolitan Counties in New Mexico

	Rural, n (%)	Metropolitan, n (%)	p
Age			
Mean	56.8 years	53.3 years	0.02
<35	2 (2.4)	12 (6.4)	
35 to 44	17 (20.2)	53 (28.2)	
45 to 54	16 (19.0)	39 (20.7)	
55 to 64	23 (27.4)	39 (20.7)	
65+	26 (31.0)	45 (23.9)	
Gender			
Male	38 (54.3)	67 (38.7)	0.04
Female	32 (45.7)	106 (61.3)	
Race and ethnicity			
Non-Hispanic White	48 (71.6)	92 (61.3)	0.19
Other	19 (28.4)	58 (38.7)	
Size of practice group			
1 physician	12 (21.8)	18 (15.0)	0.0003^*^
2 physicians	9 (16.4)	6 (5.0)	
3 to 4 physicians	16 (29.1)	17 (14.2)	
5 to 9 physicians	8 (14.5)	20 (16.7)	
10 or more physicians	10 (18.2)	59 (49.2)	
Scope of practice			
Obstetrics and gynecology	80 (95.2)	180 (95.2)	0.44
Obstetrics only	3 (3.6)	3 (1.6)	
Gynecology only	1 (1.2)	6 (3.2)	
Hours worked per week			
<40	0 (0.0)	20 (25.0)	0.002^*^
≥40	38 (100.0)	60 (75.0)	
Weeks worked per year			
<40	7 (18.9)	12 (14.8)	0.77
≥40	30 (81.1)	69 (85.2)	

^*^ Indicates significant *p* values for one-tailed *t*-test (age) or chi-square test (all others) at *p* < 0.0045 (the Bonferroni-corrected threshold).

In a separate analysis, all 27 board-certified ob-gyn subspecialists practiced in Bernalillo County. Driving distances from hospitals in the primary town of rural counties to UNM subspecialists' offices ranged from 34 to 497 kilometers. Another separate analysis of the 24 ob-gyn residency graduates disclosed that none chose to work in rural New Mexico from 2016 to 2019. Each chose to practice in either Bernalillo County or San Juan County (6), move to another state (12), or pursue fellowship training (6).

## Discussion

The number of general ob-gyns is often inadequate to address the health care of women in rural areas. To better address this workforce issue, we undertook an evaluation to determine whether there were differences in demographics and practices between ob-gyns in rural and metropolitan settings. Our findings in New Mexico, a large rural state, suggest that ob-gyns work in rural counties where there is a nearby hospital offering maternity care and where there are no subspecialists. Compared with general ob-gyns in metropolitan counties, those in rural settings tended to be older and non-Hispanic white males. Practice group sizes were smaller, and no ob-gyn reported to work part time (<40 hours per week).

Recruitment and retention can be challenging. As learned in primary care specialties, recruitment can begin before or during medical school.^14^ A BA/MD program developed by the UNM School of Medicine aims to accept high school students culturally representative of their rural communities in hopes they will practice in these underserved regions after completing medical training.^15^ The medical school has identified factors associated with minority students' success and students' future practice in rural and underserved communities, including community rootedness through ethnicity, personal history, longitudinal experience, and mentoring as well as specialized curriculum and academic support such as tutoring and advising. The single ob-gyn residency program at the UNM has not offered elective training in rural counties. It would be worthwhile to consider elective clinical rotations for ob-gyn residents in nonmetropoitan communities, since residency graduates did not choose to practice in rural New Mexico counties.

Physician shortages may be partially rectified by identifying and emphasizing factors that make a rural practice appealing to general ob-gyns. Medical professionals raised in a rural community are more likely to return.^16^ Minority status, public schools and housing quality, access to childcare, spousal employment and satisfaction, distance from family or friends, call schedules, work hours, time-off flexibility, and quality of local hospital and nursing care are all important.^17^ Job sharing arrangements through part-time retirement could allow senior ob-gyns to mentor less experienced workers while minimizing additional overhead costs.^18^

It is imperative to anticipate retirements of the predominantly male ob-gyns. This represents a particular challenge in ob-gyn, since most residency graduates are female.^19^ In their study of practice characteristics contributing to professional satisfaction and rural commitment among female physicians, Hustedde et al. found relationships with interpractice and external colleagues to be most important both personally and professionally.^20^ Links to both local and remote specialist colleagues as approachable resources were crucial. A collaborative working environment, fostered by helpful and empathetic partners, is essential to retention of rural female physicians.

Financial incentives to recruit and retain ob-gyns in nonmetropolitan areas are unlikely alone to suffice.^21^ We were unable to determine in this study which physicians received any loan repayment. Professional liability costs and pre-existing medical school debt were not strong influencers of rural practice in one study including ob-gyns.^22^ Nonetheless, federal and state programs offering loan forgiveness, small business incentives, subsidized housing, and scholarships to medical school in exchange for a commitment to practice in the students' or residents' home state or rural community have been developed, although their long-term efficacy is unclear.^23,24^

Certain limitations of our mandated survey deserve attention. Responses were self-reported. Respondents could choose to not answer certain questions due to factors such as survey length, ambiguity, or a desire for privacy. National questionnaires targeting medical students and residents in ob-gyn training may be insightful to determine what interventions might be effective in correcting the current trends. Our study did not examine recruitment or contributions of physician extenders in providing more local care. Responses did not permit knowing whether gynecologic surgeries were performed at hospitals with no maternity service, although we suspect that this was not possible at critical access hospitals in certain rural counties. In addition, we could not assess to what degree metropolitan ob-gyns were contributing to care of rural women through staffing outreach clinics or telemedicine. Lastly, this study did not consider the role of primary care providers who can provide uncomplicated office gynecology services. Using our experience with maternal transfers, we are unaware of any family physicians providing obstetric care in our rural counties during this period.

Future research should examine whether knowing more about demographic and practice characteristics of general ob-gyns in rural counties will have an impact on recruitment and retention. As provider attrition occurs in underserved areas, professional isolation with a lack of access to subspecialty care may accelerate the urgency of this work. Physician mobility and earlier retirement may further compromise the availability of women's health care in rural areas.^25^ Movement to smaller communities by the preponderance of ob-gyns practicing in saturated metropolitan markets may indirectly aid in solving the problem. Locum tenens and outreach by subspecialists in centrally located metropolitan areas are essential through provision of on-site care or consultation and telemedicine.^26^ Lastly, the challenges of insufficient ob-gyn availability represent an opportunity to partner with family physicians who would consider providing routine prenatal care with eventual delivery at a metropolitan hospital with more comprehensive services to maximize maternal and newborn safety.^5,27^

## Conclusions

Attracting and retaining doctors in rural settings is complex. This study of mandated responses by general ob-gyns in a large rural state offers insights into rural practitioners. General ob-gyns in New Mexico's rural counties practiced full time in smaller groups at hospitals with a maternity center. Rural ob-gyns tended to be older and non-Hispanic males. Although financial aid and loan repayment programs are important for physician recruitment to rural areas, other factors may be more important to recruitment and retention. More student and resident training opportunities in rural communities may aid in recruiting resident graduates. Some isolation of general ob-gyns in rural areas may be relieved by stronger relationships with subspecialists through different forms of outreach and care sharing and by support and mentoring of the mostly female residents who may be their junior partners as they graduate and seek their first jobs.

## Abbreviations Used

HRRC: Human Research Review Committee
ob-gyns: obstetrician–gynecologist physicians
RLD: Regulation and Licensing Department
UNM: University of New Mexico
